# Quality of Life and Well-Being of Syrian Refugees: Insights from Spain and Jordan

**DOI:** 10.3390/bs16101885

**Published:** 2026-10-13

**Authors:** Dalia Al-Hourani, Mahmoud Al-Wriekat, Jorge Juan Alvarado-Omenat, Daniel García-García, Rocío Llamas-Ramos, Inés Llamas-Ramos

**Affiliations:** 1 Department of Occupational Therapy, Faculty of Applied Medical Sciences, Jordan University of Science and Technology, Irbid 22110, Jordan; daliaalhourani@gmail.com; 2 Clinical Psychology, Faculty of Arts and Sciences, Al-Ahliyya Amman University, Amman 19111, Jordan; 3 FisioSport Salamanca, C. Doce de Octubre, 37008 Salamanca, Spain; 4 Instituto de Investigación Biomédica de Salamanca (IBSAL), Paseo San Vicente, 182, 37007 Salamanca, Spaininesllamas@usal.es (I.L.-R.); 5 Department of Nursing and Physiotherapy, Universidad de Salamanca, Avd. Donantes de Sangre s/n, 37007 Salamanca, Spain; 6 University Hospital of Salamanca, 37007 Salamanca, Spain

**Keywords:** quality of life, physical quality of life, psychological quality of life, social quality of life, environmental quality of life, Syrian refugees, Spain, Jordan, host countries

## Abstract

Syrian refugees face numerous challenges, including conflict, displacement, family separation, human rights violations, limited access to basic needs, and exposure to violence and torture. These experiences significantly affect their physical, psychological, social, and environmental quality of life (QoL). The aim of this study was to assess the QoL of Syrian refugees in Spain and Jordan and to examine the association between sociodemographic characteristics, living conditions, and experiences of discrimination with different QoL domains. This study used a cross-sectional correlational design in Syrian refugee centers, homes, and camps across Spain and Jordan. A convenience sample of Syrian refugees with confirmed status in the past ten years was recruited. Demographic data were collected via a demographic form, and the World Health Organization Quality of Life—Brief Questionnaire (WHOQOL-BREF) was used to assess their quality of life. Data analysis included descriptive statistics, Chi-square tests, one-way ANOVA, and independent *t*-tests. The two hundred participants were predominantly aged 18–29 (43%), female (52.5%), married (51.5%), with secondary school education (46.5%), and unemployed (70%), with low monthly family income (60.5%). While 69% experienced low levels of discrimination, 33% were satisfied with their health, and 47.5% rated their QoL as neutral. Refugees in Spain reported higher QoL across physical, psychological, social, and environmental domains compared to those in Jordan (*p* < 0.05). The QoL was related to socio-demographic factors: higher among married participants (*p* = 0.044), urban residents (*p* < 0.001), and recent immigrants (<5 years, *p* < 0.001), but lower among those with low income (*p* = 0.011). Gender differences showed that males had better physical (*p* = 0.001) and psychological (*p* = 0.032) QoL than females. Lower discrimination levels and employment were associated with higher QoL across multiple domains. This study highlights the lasting association of the Syrian conflict on refugees’ QoL, emphasizing the need for holistic healthcare that addresses physical, psychological, social, and environmental challenges both before and after displacement. Findings call for comprehensive support systems, including early mental health interventions, anti-discrimination efforts, and community inclusion programs, to enhance refugees’ resilience and integration. The study identifies specific subgroups at greater risk of lower QoL—including women, older adults, those with low income, the unemployed, camp residents, and those experiencing discrimination—providing actionable insights for policymakers to target resources and interventions effectively. Tailored rehabilitation and psychological care are essential for empowering refugees to rebuild their lives and fostering social cohesion in host communities.

## 1. Introduction

Refugees endure persecution, conflict, displacement, family separation, human rights violations, and limited access to essential needs such as food, water, and shelter (Abu-Awwad et al., 2020; Klingberg et al., 2020). Many are also subjected to physical violence, sexual abuse, and torture. The displacement of Syrians remains one of the world’s largest and most protracted refugee situations. According to the United Nations High Commissioner for Refugees (UNHCR) Syria Regional Refugee Response, more than 4.7 million Syrian refugees were registered across the main countries and regions of asylum, including 1.79 million registered by UNHCR in Egypt, Iraq, Jordan, and Lebanon, 2.87 million registered by the Government of Türkiye, and more than 43,000 registered in North Africa (UNHCR, 2025).

Studies show that refugees generally experience poor quality of life (QoL), which correlates with the severity of mental health issues (Leiler et al., 2019). Moreover, war-associated events can negatively affect refugees and persist for years, destructively influencing their QoL even after hostile acts have ceased (Akinyemi et al., 2012; Matanov et al., 2013). Jordan has been one of the main countries of refuge for Syrians since the beginning of the Syrian conflict and has hosted a large Syrian refugee population for more than a decade. Syrian refugees in Jordan live both inside and outside formal refugee camps, with the majority residing in host communities (Obi, 2021). The Jordanian response has been implemented through collaboration between national authorities, UNHCR, and humanitarian and development organizations, with strategies addressing access to basic services, protection, livelihoods, self-reliance, and social resilience (UNHCR, 2025). This context is particularly relevant to QoL because socioeconomic vulnerability, employment opportunities, housing conditions, access to healthcare, and reliance on humanitarian assistance may shape refugees’ physical, psychological, social, and environmental well-being (Stillman et al., 2022). In Jordan, for example, a study found that 87% of Syrian refugees living outside camps earned less than 300 JD monthly, 63.6% had low educational levels, and nearly half lived in small, overcrowded households. Despite receiving humanitarian aid, they reported poor QoL across physical, psychological, environmental, and social domains (Alwedyan, 2022). Refugees living in camps in Jordan reported lower QoL than those outside, although camps provide a safety net for those unable to leave (Obi, 2021).

In Germany, a study of 114 Syrian refugees showed that older age was linked to lower QoL, especially in psychological and physical health, with refugees in housing having better mental health than those in camps. Long-term refugees also reported better psychological outcomes than recent arrivals (Al Masri et al., 2021). Similarly, in Sweden, depression and anxiety affected 61.9% of Syrian refugees, with younger age, male gender, social support, and cohabitation being linked to better health-related QoL (Gottvall et al., 2020).

Despite the growing body of research on Syrian refugee QoL in individual host countries, no study to date has directly compared the QoL of Syrian refugees residing in a neighboring middle-income country (Jordan) with those in a European high-income country (Spain). This comparison provides a unique opportunity to examine how refugee quality of life may vary across two markedly different host-country contexts, characterized by distinct economic conditions, social welfare systems, migration policies, and integration opportunities. Such a comparison is critical because the host-country context—including asylum policies, economic opportunities, social acceptance, and living conditions—may shape refugee QoL in distinct ways (Iglesias-Martínez & Estrada, 2018; Bris & Bendito, 2017).

To move beyond a purely descriptive approach, this study adopts an integrated stress-ecological perspective. According to Miller and Rasmussen’s Ecological Model of Refugee Adjustment, QoL is associated not only with prior war-related trauma but also with ongoing post-displacement challenges (including discrimination, social isolation, and material scarcity), as well as the coping strategies and resources available to individuals (Miller & Rasmussen, 2017). This model differentiates between risk factors that negatively affect QoL (such as low income, unemployment, and extended camp stays) and protective factors that help mitigate these difficulties (such as employment, family support, and stable housing).

Guided by this framework, the present study addresses the following research question: To what degree are sociodemographic characteristics, living conditions, and perceived discrimination associated with QoL among Syrian refugees, and how do these patterns differ between Spain and Jordan? We propose the following hypotheses: (1) Due to variations in economic prospects and reception systems, refugees in Spain will report higher QoL across all four WHOQOL-BREF domains than those in Jordan. (2) In both countries, resilience factors (including male gender, marriage, urban residence, full-time employment, higher income, shorter time since immigration, and low perceived discrimination) will be associated with higher QoL. (3) In contrast, risk factors (including low income, unemployment, camp residence, longer immigration duration, and perceived discrimination) will be associated with lower QoL.

Accordingly, the present study has two primary aims. First, we describe and compare the physical, psychological, social, and environmental QoL of Syrian refugees in Jordan and Spain using the WHOQOL-BREF. Second, we examine the associations between sociodemographic characteristics, living conditions, employment status, income, time since immigration, and perceived discrimination with each QoL domain, while interpreting these factors through a risk-and-resilience lens. By addressing these aims, this study seeks to inform tailored interventions and policies that address the specific needs of Syrian refugees in different host country settings.

## 2. Materials and Methods

### 2.1. Study Design and Sampling

This study employed a cross-sectional correlational design to assess and compare QoL among Syrian refugees in Jordan and Spain. This design was chosen to gather valuable data to inform the planning and allocation of mental health services and ultimately enhance refugees’ QoL. Syrian nationals with confirmed refugee status within the past ten years were recruited through multiple methods, including invitations at participating sites, social media posts, and referrals from other refugees (snowball sampling). The final sample comprised 200 participants, with 100 respondents recruited in each country (Jordan and Spain). Recruitment followed a consecutive sampling approach and continued until the target sample size was reached. This sample size was determined to be sufficient based on statistical power calculations and comparable studies in refugee mental health research. It allows for robust comparative analyses between the two countries while remaining feasible given the challenges of accessing refugee populations. Although larger samples could provide additional granularity, 200 participants represent a balance between statistical reliability and practical constraints.

### 2.2. Data Collection

Participants were recruited from various refugee camps, centers, residences, communities, and villages in Jordan and Spain. Data collection took place between February 2023 and February 2024. Each participant spent approximately 45 min completing the self-reported questionnaires in a single session.

All study materials were provided in Arabic using validated Arabic-language versions of the instruments. Participants completed the questionnaires independently, and no interpreter support or additional translation assistance was required during data collection. No missing values were found in the dataset. All 200 participants completed all items of the demographic form and all 26 items of the WHOQOL-BREF. Completeness was ensured through real-time verification by research assistants during data collection.

### 2.3. Study Tools

#### 2.3.1. Demographic Characteristics

A structured demographic form was developed based on prior refugee quality of life literature and input from local refugee service organizations. The form collected information on nationality, gender, age, marital status, educational level, living arrangement, employment status, monthly family income, years since immigration, and perceived discrimination.

Nationality and refugee status were verified through official Syrian documentation, with eligibility requiring confirmed refugee status within the past ten years. Gender was recorded as male or female. Age was recorded in years and subsequently categorized into five groups: 18–29, 30–39, 40–49, 50–59, and 60 years or older. Marital status options included married, single, widowed, and divorced. Educational level was assessed using eight categories: illiterate, elementary, secondary, diploma, bachelor’s, master’s, and doctoral.

Living arrangement was assessed using an item adapted from UNHCR living condition classifications, with response options including big cities, large towns, smaller villages or rural areas, low social support (defined as a single-person household with no local network), and camps. Employment status was categorized as not employed, part-time employment (fewer than 35 h per week), full-time employment (35 or more hours per week), or retired. Monthly family income was self-rated as low, middle, or high relative to each country’s cost of living and refugee assistance levels. This subjective approach was chosen because absolute income comparisons between Jordan and Spain are not meaningful given the different economies and support systems. Years since immigration was categorized as 10 years, 9–5 years, or less than 5 years.

Perceived discrimination was measured using three self-developed single-item questions based on qualitative work with Syrian refugees and guidance from local non-governmental organizations. The first item asked, “In the past 12 months, how often have you felt discriminated against because of your refugee status or nationality?”, with response options of never, rarely, sometimes, often, or always. Responses were coded as low (never or rarely), medium (sometimes), or high (often or always). The second item asked, “In what areas have you experienced discrimination? (Select all that apply)”, with options including employment, housing, healthcare, education, public services, and others; this item was used for descriptive purposes only. The third item asked, “How much has discrimination affected your daily life and well-being?”, with responses ranging from not at all to extremely, and was also used for descriptive purposes only. These single-item measures were chosen for pragmatic reasons: to minimize participant burden, as total survey time was capped at 45 min including the WHOQOL-BREF, and to align with partner organization requests for a rapid, actionable screening tool. The primary item measuring frequency of discrimination was used in statistical analyses as a three-level categorical variable. Full wording and justification are provided here to ensure transparency.

#### 2.3.2. World Health Organization Quality of Life—Brief Questionnaire (WHOQOL-BREF)

The WHOQOL-BREF is one of the best-known tools developed for cross-cultural comparisons of QoL and is available in more than 40 languages, including Arabic (World Health Organization Quality of Life Group, 1994). The WHOQOL-BREF contains 26 items that cover four different domains of QoL, (1) physical QoL, which consists of seven items; (2) psychological QoL, which consists of six items; (3) social QoL, which consists of three items; and (4) environmental QoL, which consists of eight items, so that the total scores for the questionnaire range from (0–130) and (0–100) for each dimension separately, as the minimum score is zero and the maximum score is 100. The WHOQoL-BREF uses a 5-point Likert scale from 1 to 5 for each item, and it contains two main items that ask about the general QoL and are not calculated in the QoL rate, which are items No. 1 and 2, and three reversed items, No. 3, 4, and 26 (The WHOQOL Group, 1995, 1998). The study by Skevington et al. (2004) showed that the WHOQoL-BREF is valid and reliable. This scale was applied to the participants and scored by the researcher using the Arabic version (Ohaeri & Awadalla, 2009).

For the purpose of the analyses, overall quality of life was operationalized as a categorical variable using a cut-off score of 50 on the 0–100 transformed total scale. Participants with a total WHOQOL-BREF score of 50 or above were classified as having ‘High QoL,’ while those with a total score below 50 were classified as having ‘Low QoL.’ This cut-off was selected because a score of 50 represents the midpoint of the 0–100 scale and has been used in previous refugee and immigrant health research as a threshold for distinguishing poorer from better quality of life (insert references if available). The categorical operationalization was used for the descriptive comparison to facilitate interpretation of the distribution of sociodemographic characteristics across lower and higher overall QoL groups.

### 2.4. Ethical Considerations

Ethical approval for the study was granted by the Research Ethics Committee of the University of Salamanca (protocol code 958—26 May 2023). Participation was entirely voluntary, with participants free to withdraw at any point. After receiving a thorough explanation of the study, participants signed a consent form detailing the study’s purpose, potential risks, and researcher information. Personal data remained confidential, and participants were identified by ID numbers. While the study did not pose health risks or involve any treatment, some questions could have caused negative feelings or emotional discomfort. To mitigate this, psychological support was made available, and all participant concerns were fully addressed.

### 2.5. Data Analysis

Statistical analyses were conducted using the Statistical Package for the Social Sciences (SPSS) version 25.0. Categorical variables, including demographics and QoL, were described using frequencies and valid percentages. These were expressed as numbers and percentages. The WHOQOL-BREF domain scores were used to descriptively characterize the multidimensional QoL profile of participants in each country. Domain-specific scores were not prespecified as separate hypothesis tests in the analysis plan and were therefore not subjected to additional post hoc inferential comparisons. Categorical variables were compared between the Spanish and Jordanian groups using the chi-square test, whereas continuous variables were compared using the independent-samples *t*-test. The normality of the data was assessed through histograms and the Shapiro–Wilk test, and confirmed by the Kolmogorov–Smirnov test. For group comparisons of continuous variables, independent *t*-tests and one-way ANOVA were applied, as the data followed a normal distribution. A *p*-value of less than 0.05 was considered statistically significant (α = 0.05). For one-way ANOVA with statistically significant results (*p* < 0.05), post hoc comparisons were performed using Fisher’s Least Significant Difference (LSD) test to identify specific group differences. Where overall ANOVA was significant, but no pairwise LSD comparisons reached statistical significance, this is explicitly noted in the results.

Two operationalisations of quality of life were used in this study. First, for the descriptive comparison, overall QoL was treated as a categorical variable (Low vs. High) using a cut-off of 50 on the 0–100 transformed total scale. This categorization was used solely to compare the distribution of sociodemographic and relevant characteristics between participants with lower versus higher overall QoL and to facilitate interpretability of group differences. Second, for all subsequent analyses (the four WHOQOL-BREF domain scores (physical, psychological, social, and environmental) were analyzed as continuous variables using independent-samples *t*-tests and one-way ANOVA. This approach was chosen because continuous domain scores preserve the full variability of the data, provide greater statistical power, and avoid the loss of information associated with dichotomization. The categorical operationalization was therefore limited to the descriptive analysis, while the continuous operationalization was used for all inferential analyses examining associations between sociodemographic factors and QoL domains.

## 3. Results

Table 1 provides an overview of the socio-demographic and relevant characteristics of the participants, with 100 residing in Jordan and 100 in Spain. Of the total sample, 43% were aged 18–29 years. About half of the participants were married (51.5%), female (52.5%), and had a secondary school education (46.5%). Most participants resided in urban areas or nearby municipalities (60%), were unemployed (70%), and had a low monthly family income (60.5%). Additionally, 69% reported low levels of discrimination, while 36.5% had immigrated to host countries 10 years ago, 33% were satisfied with their health, and 47.5% rated their QoL as neutral (neither poor nor good).

Statistical comparisons between Jordan and Spain for categorical variables were conducted using chi-square tests (Table 1). Independent *t*-tests were used to compare continuous QoL domain scores between two groups (e.g., gender, country of residence), while one-way ANOVA with post hoc LSD tests was used for comparisons involving three or more groups (e.g., age, income, years of immigration). All statistically significant findings (*p* < 0.05) are indicated in the tables.

Values are presented as mean ± SD for continuous variables and n (%) for categorical variables. Between-group comparisons were performed using the independent-samples *t*-test for continuous variables and the chi-square test for categorical variables.

In Jordan, most participants were male (60%), single (69.6%), most had immigrated 10 years ago (89%), and 57.6% were satisfied with their health. Notably, all participants living in refugee camps were based in Jordan. In contrast, in Spain, most participants were female (59%), married (67%), and residing in large cities (70.8%). Most participants who had immigrated less than five years ago were in Spain (93%), and 70% of those highly satisfied with their health were from Spain.

### 3.1. Comparison of QoL Between Spain and Jordan

**Figure 1:**
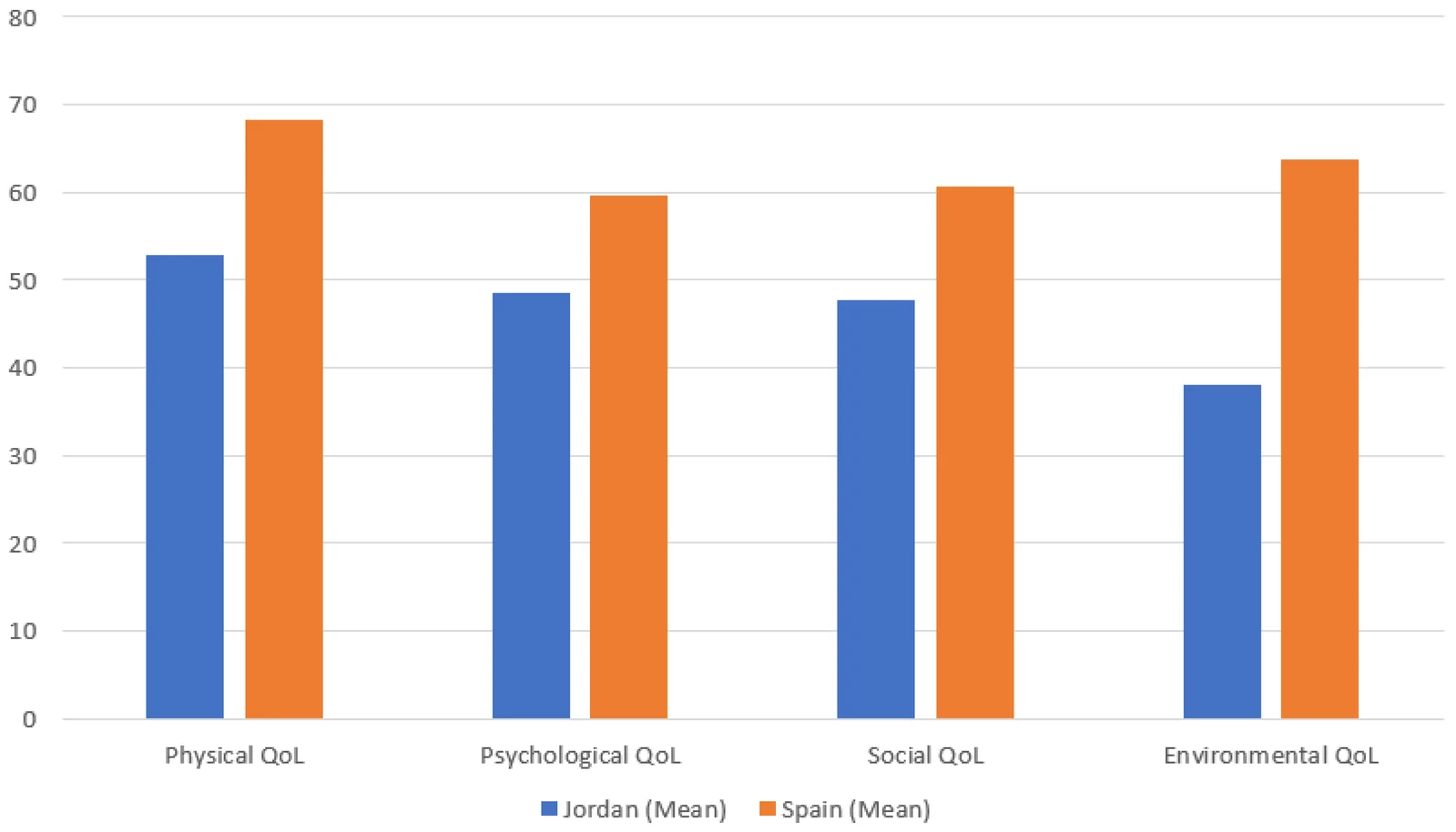
Bar Chart Comparing QoL Domains Between Spain and Jordan.

A total of 200 Syrian adults aged 18 and above participated in the study, completing the questionnaires. Figure 1 presents the mean WHOQOL-BREF scores across the four QoL domains for participants from Spain and Jordan. Overall, participants from Spain showed higher mean QoL scores than participants from Jordan across the assessed domains. These differences were complemented by the categorical distribution of QoL presented in Table 2. Statistically significant differences in the distribution of QoL categories were observed between the two countries for physical QoL (*p* < 0.001), psychological QoL (*p* < 0.001), social QoL (*p* = 0.023), and environmental QoL (*p* < 0.001).

For physical QoL, participants from Jordan were more frequently classified in the very poor, poor, and average categories, whereas participants from Spain were more frequently classified in the good and excellent categories. For example, 57.6% of participants classified as having good physical QoL and 80.5% of those classified as having excellent physical QoL were from Spain. A similar pattern was observed for psychological QoL, with 88.9% of participants classified as having excellent psychological QoL being from Spain, compared with 11.1% from Jordan. For environmental QoL, the difference was particularly pronounced, with 88.9% of participants classified as having very poor environmental QoL and 78.7% of those classified as having poor environmental QoL being from Jordan, whereas 96.6% of those classified as having excellent environmental QoL were from Spain. The distribution of social QoL categories also differed significantly between countries, although the magnitude of the difference was smaller than for the other domains.

Table 2 represents the QoL among adult Syrian refugees overall and by residency. Overall, over half of the participants reported between average (26.5%) and good (33%) physical QoL, between average (41%) and good (22.5%) psychological QoL, between average (35%) and good (25%) social QoL, and between poor (23.5%) and average (36.5%) environmental QoL. In Jordan, scores were lower than in Spain in physical QoL (average (77.4%) vs. good (57.6%) to excellent (80.5%), *p* = 0.000), psychological QoL (average (63.4%) vs. average (36.6%), good (53.3%) to excellent (88.9%), *p* = 0.000), social QoL (average (58.6%) vs. average (41.4%) to good (58%), *p* = 0.023), and environmental QoL (poor (78.7%) to average (54.8%) vs. average (45.2%), *p* = 0.000).

These domain-level differences are presented descriptively to characterize the multidimensional QoL profiles of the two groups.

### 3.2. Association Between Resilience Factors (Employment, Urban Living, Marriage, Recent Immigration) and QoL

Table 3 shows the QoL according to the sociodemographic and relevant characteristics among adult Syrian refugees overall and by residency. Overall, married participants showed higher QoL levels than unmarried participants (*p* = 0.044). Higher QoL was also observed among participants living in large cities (*p* < 0.001) and among those who had immigrated within the previous five years (*p* < 0.001). Lower QoL was observed among participants reporting low family income (*p* = 0.011). In Jordan, QoL was low among those who had low monthly family income levels (68.3%, *p* = 0.007). In Spain, QoL was high among individuals living in big cities (87.1%, *p* = 0.001).

### 3.3. Association Between Risk Factors (Low Income, Unemployment, Camp Residence, Discrimination) and QoL

Table 4 compares the differences between the participants’ sociodemographic and relevant characteristics based on their QoL, regardless of their country of residence. An independent-samples *t*-test was conducted to compare the psychological QoL between male and female genders. There was a significant difference in the scores for males (M = 56.80, SD = 19.975) and females (M = 50.44, SD = 21.472); t(198) = 2.163, *p* = 0.032. These results suggest that Syrian adult male refugees experience higher levels of psychological QoL compared to their female counterparts.

A one-way ANOVA revealed that there was a statistically significant difference in environmental QoL scores between the employment status groups (F(3, 196) = 2.875, *p* = 0.037). An LSD test for multiple comparisons found that the mean value of environmental QoL was different between not employed and full-time employment (*p* = 0.020, 95% CI = [−21.57, −1.88]), and between part-time and full-time employment (*p* = 0.007, 95% CI = [−27.84, −4.48]). Also, a one-way ANOVA revealed that there was a statistically significant difference in psychological QoL scores between the discrimination groups (F(2, 197) = 3.418, *p* = 0.035). An LSD test for multiple comparisons found that the mean value of psychological QoL was different between low and medium discrimination (*p* = 0.011, 95% CI = [1.99, 15.10]).

In addition, there was a statistically significant difference in physical QoL scores between the years of immigration groups (F(2, 197) = 9.514, *p* = 0.000). An LSD test for multiple comparisons found that the mean value of physical QoL was different between 10 years and less than 5 years of immigration (*p* = 0.000, 95% CI = [−21.89, −8.25]). Also, a one-way ANOVA revealed that there was a statistically significant difference in social QoL scores between the years of immigration groups (F(2, 197) = 4.989, *p* = 0.008). An LSD test for multiple comparisons found that the mean value of social QoL was different between 10 years and less than 5 years of immigration (*p* = 0.007, 95% CI = [−19.90, −3.14]). Moreover, a one-way ANOVA revealed that there was a statistically significant difference in environmental QoL scores between the years of immigration groups (F(2, 197) = 22.178, *p* = 0.000). An LSD test for multiple comparisons found that the mean value of environmental QoL was significantly different between 10 years and less than 5 years of immigration (*p* = 0.000, 95% CI = [−30.56, −16.58]).

In this line, the one-way ANOVA revealed that there was a statistically significant difference in physical QoL scores between the monthly family income groups (F(2, 197) = 3.282, *p* = 0.040). Also, a one-way ANOVA revealed that there was a statistically significant difference in psychological QoL scores between the monthly family income groups (F(2, 197) = 4.124, *p* = 0.018). An LSD test for multiple comparisons found that the mean value of psychological QoL was different between low and high monthly family income (*p* = 0.007, 95% CI = [−49.19, −7.81]). Also, a one-way ANOVA revealed that there was a statistically significant difference in environmental QoL scores between the monthly family income groups (F(2, 197) = 9.941, *p* = 0.000). An LSD test for multiple comparisons found that the mean value of environmental QoL was significantly different between low and high monthly family income (*p* = 0.001, 95% CI = [−56.52, −14.13]).

### 3.4. Country-Specific Patterns of QoL-Associated Factors

Table 5 compares the differences in participants’ sociodemographic and relevant characteristics based on their QoL, categorized by their country of residence.

#### 3.4.1. Jordan Subgroup Analyses

In Jordan, a one-way ANOVA revealed a statistically significant difference in physical QoL scores between the age groups (F(4, 95) = 3.211, *p* = 0.016). Post hoc LSD comparisons showed that physical QoL was significantly higher among participants aged 18–29 compared to those aged 40–49 (*p* = 0.003, 95% CI [4.96, 23.31]), higher among those aged 30–39 compared to 40–49 (*p* = 0.002, 95% CI [5.65, 25.67]), and higher among those aged ≥60 compared to 40–49 (*p* = 0.034, 95% CI [−49.82, −1.93]).

Similarly, a one-way ANOVA revealed a statistically significant difference in psychological QoL scores between the age groups (F(4, 95) = 2.508, *p* = 0.046). Post hoc LSD comparisons revealed significantly higher psychological QoL among participants aged 18–29 compared to 40–49 (*p* = 0.003, 95% CI [5.15, 23.92]), and among those aged 30–39 compared to 40–49 (*p* = 0.022, 95% CI [1.82, 22.29]).

A one-way ANOVA revealed a statistically significant difference in psychological QoL scores between marital status groups (F(3, 96) = 3.799, *p* = 0.013). Post hoc LSD comparisons showed that single participants reported significantly higher psychological QoL than married participants (*p* = 0.003, 95% CI [−17.46, −3.61]).

Regarding living arrangements, a one-way ANOVA revealed a statistically significant difference in psychological QoL scores between living arrangement groups (F(3, 96) = 3.675, *p* = 0.015). Post hoc LSD comparisons revealed that participants living in big cities reported significantly higher psychological QoL than those in small villages (*p* = 0.001, 95% CI [6.63, 26.61]).

In relation to environmental QoL scores, there was a statistically significant difference between educational level groups (F(6, 93) = 2.915, *p* = 0.012). Post hoc LSD comparisons showed significantly higher environmental QoL among participants with master’s degrees compared to those with elementary education (*p* = 0.004, 95% CI [−47.65, −9.48]) and secondary education (*p* = 0.003, 95% CI [−45.62, −9.27]), and among those with bachelor’s degrees compared to illiterate participants (*p* = 0.016, 95% CI [−50.77, −5.34]).

Regarding employment status, a one-way ANOVA revealed a statistically significant difference in environmental QoL scores between employment status groups (F(3, 96) = 3.392, *p* = 0.021). Post hoc LSD comparisons revealed that full-time employed participants reported significantly higher environmental QoL than unemployed participants (*p* = 0.025, 95% CI [−12.93, 1.56]).

Additionally, a one-way ANOVA revealed a statistically significant difference in environmental QoL scores between monthly family income groups (F(2, 97) = 4.800, *p* = 0.010). Post hoc LSD comparisons found that environmental QoL was significantly higher among participants with middle income compared to low income (*p* = 0.017, 95% CI [−17.93, −1.81]), and among those with high income compared to low income (*p* = 0.024, 95% CI [−24.92, −3.03]).

#### 3.4.2. Spain Subgroup Analyses

In Spain, an independent-samples *t*-test was conducted to compare physical QoL between male and female participants. There was a significant difference in scores for males (M = 75.34, SD = 19.113) and females (M = 61.15, SD = 21.063); t(98) = 3.386, *p* = 0.001. These results suggest that Syrian adult male refugees experience higher levels of physical QoL compared to their female counterparts. Similarly, an independent-samples *t*-test comparing psychological QoL between male and female participants showed a significant difference in scores for males (M = 65.55, SD = 21.574) and females (M = 53.66, SD = 23.616); t(98) = 2.524, *p* = 0.013.

A one-way ANOVA revealed a statistically significant difference in physical QoL scores between marital status groups (F(3, 96) = 3.421, *p* = 0.020). Post hoc LSD comparisons showed that single participants reported significantly higher physical QoL than married participants (*p* = 0.013, 95% CI [−22.25, −2.74]) and divorced participants (*p* = 0.019, 95% CI [3.79, 41.21]).

Regarding living arrangement, a one-way ANOVA revealed statistically significant differences in physical QoL scores between living arrangement groups (F(3, 96) = 4.755, *p* = 0.004), in social QoL scores (F(3, 96) = 2.771, *p* = 0.046), and in environmental QoL scores (F(3, 96) = 4.579, *p* = 0.005). Post hoc LSD comparisons indicated that participants living in big cities reported significantly higher QoL across these domains compared to those in smaller villages and rural areas (physical: *p* = 0.002; social: *p* = 0.035; environmental: *p* = 0.001).

A one-way ANOVA revealed a statistically significant difference in psychological QoL scores between discrimination groups (F(2, 97) = 3.390, *p* = 0.038). Post hoc LSD comparisons found that participants reporting low discrimination had significantly higher psychological QoL than those reporting medium discrimination (*p* = 0.011, 95% CI [3.06, 23.46]). Similarly, a one-way ANOVA revealed a statistically significant difference in environmental QoL scores between discrimination groups (F(2, 97) = 5.666, *p* = 0.005). Post hoc LSD comparisons showed that participants reporting low discrimination had significantly higher environmental QoL than those reporting medium discrimination (*p* = 0.001, 95% CI [5.91, 23.08]).

For the remaining statistically significant ANOVA results reported in Table 3, Table 4 and Table 5, post hoc LSD comparisons revealed no statistically significant pairwise differences, and this has been indicated in the corresponding footnotes.

**Figure 2:**
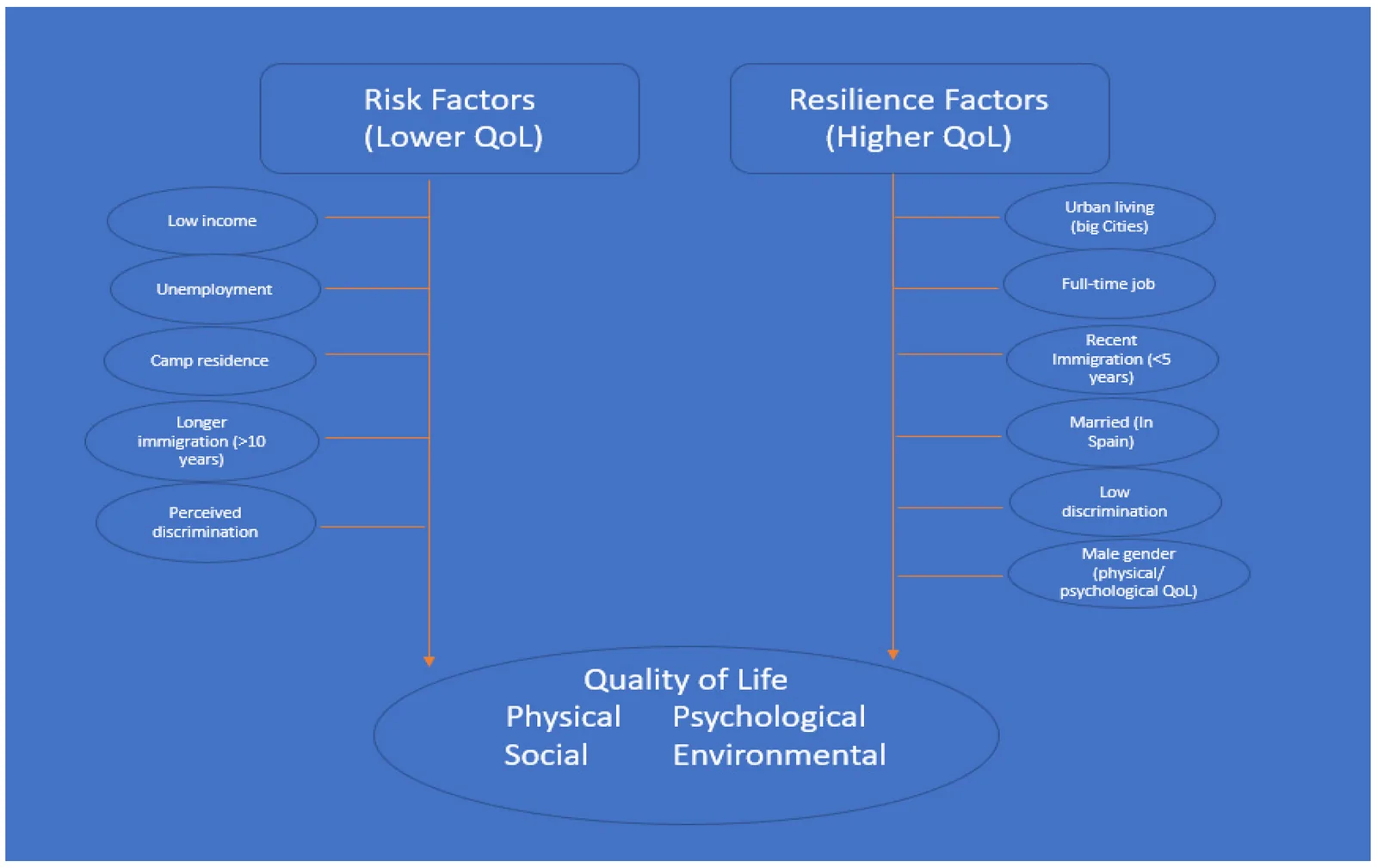
Risk and resilience factors.

Figure 2 presents a conceptual summary of the risk and resilience factors identified through bivariate comparisons. A conceptual model of risk and resilience factors associated with quality of life among Syrian refugees, based on bivariate findings from the present study. Arrows indicate direction of association; factors shown were statistically significant (*p* < 0.05) in at least two QoL domains.

## 4. Discussion

While the present study employed bivariate comparisons to document QoL differences between Jordan and Spain, interpreting these findings through a risk-and-resilience lens provides valuable integrative insight. Factors associated with lower QoL across domains—including low income, unemployment, camp residence, longer immigration duration (>10 years), and perceived discrimination—can be understood as post-displacement risk factors that erode refugee well-being. Conversely, factors associated with higher QoL—such as urban residence, full-time employment, shorter immigration duration (<5 years), and low discrimination—function as resilience factors or protective resources. Notably, some factors showed domain-specific effects: marriage was protective for psychological QoL in Spain but not Jordan, while recent immigration was consistently associated with a higher physical, social, and environmental QoL. This risk-and-resilience interpretation aligns with the ecological model of refugee adjustment (Miller & Rasmussen, 2017) and suggests that interventions should simultaneously reduce risks (e.g., poverty, discrimination) while strengthening resources (e.g., employment, urban integration). However, a limitation of our bivariate approach is that it cannot isolate the unique contribution of each factor after controlling for others; future studies should employ multivariable regression to test these pathways directly.

Most participants in the present study were unemployed, possessed secondary education qualifications, and earned low incomes. These characteristics are consistent with the broader literature indicating that the Syrian conflict led to widespread displacement, including that of academics and educators, which exacerbated educational challenges and created demands on host countries (Seydi, 2013). However, given our convenience sampling approach, these proportions should not be assumed to reflect the broader Syrian refugee population. Preceding the crisis, Syria faced educational disparities linked to regional and parental backgrounds, alongside rising dropout rates among older adolescents (Welker et al., 2021). The conflict, ongoing since 2011, has severely disrupted what were once high enrollment rates in primary and secondary education, placing immense strain on Syria’s educational system (Al Hessan et al., 2021). Syrian refugees in neighboring countries, particularly Lebanon, encountered significant obstacles to accessing education, even though it remained a high priority for families (Akesson et al., 2020).

Our findings are broadly consistent with prior research highlighting the substantial challenges Syrian refugees face in accessing education, achieving stable income, and securing employment in host countries. For example, studies conducted in Turkey have reported that only 38.6% of refugees were employed, primarily in unpaid or irregular jobs (Kayaoglu & Erdoğan, 2019), while research in Jordan found that less than 20% of refugees were engaged in work, mostly within informal or illegal sectors (Krafft et al., 2019). Direct comparisons should be made cautiously given differences in sampling and context. In the Netherlands, Syrian refugees often participate in volunteer work alongside language classes but rarely pursue employment or formal education (Miltenburg & Dagevos, 2021). Education remains a pressing concern, with limited access to basic education in Turkey (Bircan & Sunata, 2015) and low school enrollment rates reported in Jordan (Krafft et al., 2019). Key factors shaping employment opportunities include language proficiency, age, education level, and gender (Kayaoglu & Erdoğan, 2019; Miltenburg & Dagevos, 2021).

Research shows that refugees in Spain endure precarious conditions during their initial five years of integration, including economic instability, inadequate housing, and job insecurity (Iglesias-Martínez & Estrada, 2018). The Spanish refugee reception system has faced criticism for failing to adequately address these challenges (Iglesias-Martínez & Estrada, 2018; Maristany, 2020). The refugee crisis has attracted considerable media attention, with press coverage often emphasizing visual depictions of the conflict (Guijarro & Cuba, 2018). Spain’s approach of delegating its refugee system to NGOs through subsidies has been deemed both unsustainable and ineffective in responding to the rising demand for asylum (Bris & Bendito, 2017).

At the same time, in the present sample, participants reported QoL scores ranging from average to good in the physical, psychological, and social domains, while environmental QoL scores varied from poor to average. These findings reflect the experiences of our specific sample and may not be representative of all Syrian refugees in Jordan or Spain. Among participants in our study, those residing in Jordan reported lower physical, psychological, social, and environmental QoL compared to those residing in Spain. While these differences were statistically significant, they may reflect the specific characteristics of our sample, including differences in living arrangements, employment status, and duration of displacement between the two subsamples. Within our sample, married individuals, those living in large cities, and recent immigrants (less than five years) reported higher QoL, whereas QoL was lower among those with low income. These associations were identified through bivariate analyses and should be interpreted as exploratory; multivariable analyses are needed to determine whether these factors independently predict QoL after controlling for potential confounders. Physical QoL was higher among those living in big cities and significantly lower among those with low income and those who had been immigrants for ten years.

Contrary to our findings, research in Jordan has reported poor to average QoL across physical, psychological, social, and environmental domains, with scores below 50 on a 100-point scale (Alwedyan, 2022; Nashwan et al., 2022). Comparatively, Syrian refugees in Germany showed low QoL scores across all domains, particularly in social relations and psychological aspects (Al Masri et al., 2021). Factors influencing QoL included marital status, income, employment, and health conditions (Cevik et al., 2022). Education level was also significant, with higher education associated with better QoL among older refugees (Cevik et al., 2022). In Germany, asylum duration and housing type were linked to psychological and social relationship domains (Al Masri et al., 2021). Despite support provided in host countries, Syrian refugees continue to face challenges in maintaining their overall QoL (Alwedyan, 2022).

Psychological QoL was higher among males, those living in big cities, and those exposed to little or no discrimination, but lower among individuals with low income. Social QoL was higher among those living in big cities and lower among those who had been immigrants for ten years. Environmental QoL was higher among those living in big cities, those with full-time employment, and those who had been immigrants for less than five years, but lower among individuals with low income.

In Jordan, QoL was lower among individuals with low income. Physical QoL was lower among those aged 40 to 49 years. Psychological QoL was lower among individuals aged 40 to 49 years and those living in villages, but higher among single individuals. Environmental QoL was lower among those residing in camps, the illiterate, the unemployed, and those with low income. Conversely, it was higher among individuals with higher education levels.

Previous studies have also found that Syrian refugees face significant challenges in QoL across various domains. Research in Jordan, Germany, and Turkey has shown that Syrian refugees generally score lower on QoL measures compared to host populations (Alwedyan, 2022; Al Masri et al., 2021; Nashwan et al., 2022; Cevik et al., 2022). Factors positively associated with higher QoL include experiencing less discrimination, having full-time employment, and living in big cities (Al Masri et al., 2021). Conversely, larger family sizes and low income negatively impact QoL (Alwedyan, 2022; Nashwan et al., 2022). Psychological well-being is particularly linked to refugees scoring lower in this domain compared to host populations (Nashwan et al., 2022). However, some studies found that better housing conditions and longer asylum duration were associated with improved environmental and psychological QoL (Al Masri et al., 2021). Surprisingly, this finding contradicts our results, as poorer general, social, and environmental QoL were reported among individuals with longer immigration periods, while significantly higher QoL was observed among those residing for less than five years. This discrepancy may be explained by homesickness and ongoing conflicts in Syria, which may eventually affect the level of support provided by host countries to Syrian refugees. Nevertheless, this finding requires more detailed examination, and future studies are recommended.

In our study, Syrian refugees residing in Jordan reported mostly average scores in physical, psychological, and social QoL, but average to poor environmental QoL. One previous study in Jordan, comparing Syrian refugees to low-socioeconomic-status Jordanians, found that refugees had significantly lower scores in social relationships and psychological health but higher scores in the environmental domain (Abdo et al., 2019). Physical health scores were similar between the two groups. Factors positively influencing QoL included being married, having higher income, and being employed (Abdo et al., 2019). These findings warrant further investigation in future research.

Among participants residing in Spain, QoL was higher among those living in big cities. This finding is consistent with previous research suggesting that urban residence may facilitate access to services and employment opportunities (Al Masri et al., 2021), although our cross-sectional design precludes causal inference and our convenience sample limits generalizability. Physical QoL was higher among males, single individuals, and those living in big cities. Psychological QoL was higher among males and lower among those exposed to medium levels of discrimination. Social QoL was higher among those living in big cities. Environmental QoL was lower among those living in villages and those with low income, but higher among those exposed to no or low discrimination.

Previous studies conducted in Spain have reported that immigrants generally report poorer mental health than natives, with perceived discrimination being a significant predictor of both physical and mental health (Sevillano et al., 2014). Our findings regarding discrimination and QoL are consistent with this literature, though our single-item measure of discrimination and convenience sample warrant caution in interpretation. In Germany, Syrian refugees showed lower QoL scores compared to the general population, with asylum duration and housing influencing psychological and social domains (Al Masri et al., 2021). Syrian refugees in Kurdistan camps scored higher on social relationship QoL compared to general population norms but lower on physical and psychological domains (Aziz et al., 2014). In Sweden, Syrian refugees reported lower health-related QoL than the general population, with post-migration stressors such as high financial strain, social strain, competency strain, and discrimination significantly lowering their health-related QoL (Sengoelge et al., 2022).

The present study assessed discrimination and social support as broad, retrospective contextual factors. However, a growing body of research suggests that these processes also operate as recurring, everyday experiences that may affect well-being in more immediate and dynamic ways. For example, Krüger (2024) used ecological momentary assessment to examine everyday discrimination, co-ethnic social support, and mood changes among young adult immigrants in Germany, including Syrian refugees. This study found that within-person increases in discrimination were associated with immediate declines in mood, and that perceived social support had a consistent positive main effect on mood regardless of whether discrimination occurred. Notably, the buffering effect of social support was modest, and the level of perceived support mattered more than the ethnic origin of the support provider (intra-ethnic vs. inter-ethnic). These findings complement broader quality-of-life research by capturing the temporal dynamics of discrimination and support as they unfold in daily life. Future research could build on the present findings by incorporating ecological momentary assessment or daily diary methods to examine how everyday discrimination and social support shape quality of life among Syrian refugees in different host-country contexts.

The present findings, while exploratory, offer several actionable insights for policymakers, service providers, and stakeholders working with Syrian refugee populations. First, the identification of specific subgroups at greater risk of lower QoL—including women, older adults (particularly those aged 40–49), individuals with low income, the unemployed, camp residents, those in rural areas, and those who have experienced discrimination—can inform targeted screening and resource allocation. For example, gender-sensitive mental health services and psychosocial support programs may be particularly beneficial for female refugees, who reported lower physical and psychological QoL than their male counterparts in our sample.

Second, the consistent association between low income and poorer QoL across multiple domains underscores the importance of economic integration policies. Vocational training, microfinance initiatives, credential recognition, and formal employment pathways are concrete measures that can address poverty as a fundamental determinant of refugee well-being. The finding that full-time employment was associated with higher environmental QoL in both countries further supports the value of employment-focused interventions.

Third, the link between perceived discrimination and lower psychological and environmental QoL highlights the need for anti-discrimination legislation, intercultural awareness campaigns, and accessible mental health services for those who experience discrimination. Policymakers should consider embedding anti-discrimination measures within broader refugee integration strategies.

Fourth, the particularly poor environmental QoL reported by camp residents in Jordan suggests that improvements in camp infrastructure, housing conditions, and access to basic services should remain a priority. For refugees in Spain, where urban residence was associated with higher QoL, policies that support affordable housing and community integration in urban areas may help sustain these benefits.

Finally, the finding that recent immigrants (less than five years) reported higher QoL than those displaced for longer periods (10 years or more) suggests that early resettlement assistance may have lasting protective effects. Policymakers should consider strengthening support during the initial resettlement period, with particular attention to family support programs, language training, and community integration initiatives. While these recommendations require validation in representative samples, they provide a starting point for evidence-informed policy development and program design.

### Limitations

Several methodological limitations should be acknowledged when interpreting these findings. First, while bivariate comparisons (*t*-tests, ANOVA) were appropriate for our primary aim of documenting cross-country differences in QoL, this approach does not allow us to isolate the unique contribution of each sociodemographic or contextual factor after controlling for potential confounders. For example, the observed association between urban living and higher QoL may be partially explained by greater employment opportunities or better access to services in cities. Although the present study identified several factors associated with quality of life, the use of bivariate analyses does not allow determination of the independent contribution of each variable while controlling for potential confounders. Future studies should employ multivariable analytical approaches to better understand the relative association of socio-demographic, contextual, risk, and resilience-related factors on quality-of-life outcomes among refugee populations.

Second, the numerous group comparisons presented increase the risk of Type I error (false positives). We have not applied corrections (e.g., Bonferroni) given the exploratory and descriptive nature of the study; therefore, findings should be interpreted cautiously pending replication.

Third, perceived discrimination was assessed using self-developed single-item measures rather than a validated multi-item scale (e.g., the Everyday Discrimination Scale). This decision was pragmatic, driven by the need to limit survey length to 45 min at the request of our community partners. While our single-item measure captured the frequency and perceived impact of discrimination, it may not fully capture its multidimensional nature (e.g., chronic versus acute experiences, attributional ambiguity). Nevertheless, single-item discrimination measures have been used successfully in refugee health research and demonstrate acceptable test–retest reliability and convergent validity. Future studies should incorporate validated Arabic-language discrimination scales where feasible.

Fourth, monthly family income was measured using country-relative self-rating (low/middle/high) rather than absolute figures, which limits cross-country comparability but improves within-country interpretability. Finally, the demographic form was not independently validated, though all items were reviewed by refugee service providers and piloted with five Syrian refugees per country prior to data collection. Despite these limitations, the study provides valuable comparative insights into the QoL of Syrian refugees in two distinct host countries.

Fifth, the study employed a convenience sample of 200 Syrian refugees (100 in Jordan and 100 in Spain), recruited through invitations at participating sites, social media posts, and snowball sampling. As such, the sample cannot be considered representative of the broader Syrian refugee population in either host country. Participants who were more accessible, more engaged with services, or more willing to participate may be overrepresented. Consequently, the findings should be interpreted as exploratory and hypothesis-generating rather than definitive. Prevalence estimates and associations observed in this sample may not generalize to the wider refugee population, and caution is warranted when extrapolating these results to policy or clinical practice. Future studies should employ probability sampling methods, where feasible, to enhance representativeness and external validity.

Sixth, the dichotomization of overall QoL into ‘Low’ and ‘High’ categories using a cut-off of 50 for the descriptive analysis in Table 3 necessarily simplifies the continuous nature of quality of life and may obscure variability within each category. While this categorization was used solely to facilitate interpretation of group differences, future research should consider using continuous measures throughout or applying established cut-offs validated in refugee populations.

## 5. Conclusions

While these findings provide valuable insights into the QoL of Syrian refugees in Spain and Jordan, they should be interpreted in light of the study’s limitations, particularly the use of a convenience sample. Future research with representative samples is needed to confirm and extend these results.

This study provides insights into the enduring impact of the Syrian conflict on the quality of life and well-being of refugees, highlighting the challenges they face even years after resettlement. The findings underscore the necessity of a holistic healthcare framework that addresses the intricate interplay between physical, psychological, social, and environmental dimensions of QoL, both before and after displacement. Recognizing the structure of the pre-conflict Syrian education system and the educational barriers refugees encounter is pivotal for designing targeted interventions and policies that facilitate integration, learning, and empowerment in host countries.

From a policy perspective, this study identifies specific demographic categories of Syrian refugees who may be at greater risk of lower QoL—including women, older adults (40–49 years), those with low income, the unemployed, camp residents, those in rural areas, and those who have experienced discrimination. These findings can inform targeted screening, resource allocation, and intervention planning. For example, gender-sensitive mental health services, economic empowerment programs, anti-discrimination initiatives, and improvements in camp infrastructure are concrete measures that policymakers and service providers can prioritize.

The findings also call for comprehensive support systems that address both pre-displacement trauma and the ongoing challenges of resettlement. Early identification of needs and tailored interventions—spanning mental health, social reintegration, and environmental stability—can substantially enhance outcomes for this vulnerable population. Furthermore, this research emphasizes the critical role of anti-discrimination efforts, community inclusion, and immediate psychological support in fostering resilience and mental well-being.

To support successful adjustment and long-term mental health, this study advocates robust psychological and psychiatric care, alongside rehabilitation programs tailored to the unique needs of refugees. These initiatives are essential not only for alleviating the psychological burden of displacement but also for empowering refugees to rebuild their lives with dignity and purpose. By addressing these interconnected challenges holistically, host nations may be better equipped to support refugees in rebuilding their lives. However, confirmation of these findings in representative samples is necessary before definitive policy recommendations can be made.

## Figures and Tables

**Table 1:** The sociodemographic and relevant characteristics of the participants.

Variables	Jordan (N = 100)		Spain (N = 100)		*p*-Value	Total (N = 200)	
	n	%	n	%		n	%
Age							
18–29	50	58.1	36	41.9	0.260	86	43
30–39	28	48.3	30	51.7		58	29
40–49	16	40	24	60		40	20
50–59	4	33.3	8	66.7		12	6
≥60	2	50	2	50		4	2
Gender							
Male	57	60	38	40	0.007	95	47.5
Female	43	41	62	59		105	52.5
Marital Status							
Married	36	35	67	65	0.000	103	51.5
Single	55	69.6	24	30.4		79	39.5
Widow	5	62.5	3	37.5		8	4
Divorced	4	40	6	60		10	5
Living Arrangement							
Big cities	35	29.2	85	70.8	0.000	120	60
Large towns	10	50	10	50		20	10
Smaller villages and rural	15	78.9	4	21.1		19	9.5
Low social support	0	0	1	100		1	0.5
Camps	40	100	0	0		40	20
Educational Level							
Illiterate	2	66.7	1	33.3	0.653	3	1.5
Elementary	17	40.5	25	59.5		42	21
Secondary	45	48.4	48	51.6		93	46.5
Diploma	13	56.5	10	43.5		23	11.5
Bachelor’s	18	56.3	14	43.8		32	16
Master’s	3	75	1	25		4	2
Doctoral	2	66.7	1	33.3		3	1.5
Employment Status							
Not employed	63	45	77	55	0.013	140	70
Part-time employment	26	74.3	9	25.7		35	17.5
Full-time employment	9	40.9	13	59.1		22	11
Retired	2	66.7	1	33.3		3	1.5
Discrimination							
Low	69	50	69	50	0.750	138	69
Medium	26	48.1	28	51.9		54	27
High	5	62.5	3	37.5		8	4
Years of Immigration							
10 years	65	89	8	11	0.000	73	36.5
9–5 years	31	44.3	39	55.7		70	35
Less than 5	4	7	53	93		57	28.5
Monthly Family Income							
Low income	63	52.1	58	47.9	0.121	121	60.5
Middle income	37	49.3	38	50.7		75	37.5
High income	0	0	4	100		4	2
Quality of Life Rate							
Very poor	10	50	10	50	0.024	20	10
Poor	20	66.7	10	33.3		30	15
Neither poor nor good	52	54.7	43	45.3		95	47.5
Good	16	34.8	30	65.2		46	23
Very good	2	22.2	7	77.8		9	4.5
The Level of Satisfaction with Your Health							
Very dissatisfied	8	44.4	10	55.6	0.002	18	9
Fairly dissatisfied	17	54.8	14	45.2		31	15.5
Neither dissatisfied nor satisfied	21	70	9	30		30	15
Satisfied	38	57.6	28	42.4		66	33
Very satisfied	16	29.1	39	70		55	27.5

**Table 2:** Distribution of WHOQOL-BREF quality-of-life categories among Syrian refugees in Jordan and Spain.

Variables	Jordan (N = 100)		Spain (N = 100)		*p*-Value	Total (N = 200)	
	n	%	n	%		n	%
Quality of life *							
Physical Quality of Life							
Very poor	4	50	4	50	0.000	8	4
Poor	19	59.4	13	40.6		32	16
Average	41	77.4	12	22.6		53	26.5
Good	28	42.4	38	57.6		66	33
Excellent	8	19.5	33	80.5		41	20.5
Psychological Quality of life							
Very poor	6	46.2	7	53.8	0.000	13	6.5
Poor	18	54.5	15	45.5		33	16.5
Average	52	63.4	30	36.6		82	41
Good	21	46.7	24	53.3		45	22.5
Excellent	3	11.1	24	88.9		27	13.5
Social Quality of Life							
Very poor	12	63.2	7	36.8	0.023	19	9.5
Poor	19	55.9	15	44.1		34	17
Average	41	58.6	29	41.4		70	35
Good	21	42	29	58		50	25
Excellent	7	25.9	20	74.1		27	13.5
Environmental Quality of Life							
Very poor	16	88.9	2	11.1	0.000	18	9
Poor	37	78.7	10	21.3		47	23.5
Average	40	54.8	33	45.2		73	36.5
Good	6	18.2	27	81.8		33	16.5
Excellent	1	3.4	28	96.6		29	14.5

* World Health Organization Quality of Life—Brief Questionnaire (WHOQOL-BREF).

**Table 3:** Quality of life according to the sociodemographic and relevant characteristics of the participants based on their country of residence.

Variable	Jordan (N = 100)				*p*-Value	Spain (N = 100)				*p*-Value	Total (N = 200)				*p*-Value
	Low QoL		High QoL			Low QoL		High QoL			Low QoL		High QoL		
	n	%	n	%		n	%	n	%		n	%	n	%	
Gender															
Male	31	54.4	26	45.6	0.399	5	13.2	33	86.8	0.244	36	18	59	29.5	0.867
Female	27	62.8	16	37.2		14	22.6	48	77.4		41	20.5	64	32	
Marital State															
Married	22	61.1	14	38.9	0.160	11	16.4	56	83.6	0.210	33	16.5	70	35	0.044
Single	28	50.9	27	49.1		4	16.7	20	83.3		32	16	47	23.5	
Widow	4	80	1	20		1	33.3	2	66.7		5	2.5	3	1.5	
Divorced	4	100	0	0		3	50	3	50		7	3.5	3	1.5	
Living Arrangement															
Big cities	19	54.3	16	45.7	0.211	11	12.9	74	87.1	0.001	30	15	90	45	0.000
Large towns	4	40	6	60		4	40	6	60		8	4	12	6	
Small village	12	80	3	20		3	75	1	25		15	7.5	4	2	
Low social	0	0	0	0		1	100	0	0		1	0.5	0	0	
Camps	23	57.5	17	42.5		0	0	0	0		23	11.5	17	8.5	
Employment Status															
Not employed	41	65.1	22	34.9	0.090	15	19.5	62	80.5	0.937	56	28	84	42	0.165
Part-time	14	53.8	12	46.2		2	22.2	7	77.8		16	8	19	9.5	
Full-time	3	33.3	6	66.7		2	15.4	11	84.6		5	2.5	17	8.5	
Retired	0	0	2	100		0	0	1	100		0	0	3	1.5	
Discrimination															
Low	39	56.5	30	43.5	0.903	11	15.9	58	84.1	0.248	50	25	88	44	0.574
Medium	16	61.5	10	38.5		8	28.6	20	71.4		24	12	30	15	
High	3	60	2	40		0	0	3	100		3	1.5	5	2.5	
Years of Immigration															
10 years	38	58.5	27	41.5	0.946	2	25	6	75	0.825	40	20	33	16.5	0.000
9–5 years	18	58.1	13	41.9		8	20.5	31	79.5		26	13	44	22	
Less than 5	2	50	2	50		9	17	44	83		11	5.5	46	23	
Monthly Family Income															
Low	43	68.3	20	31.7	0.007	13	22.4	45	77.6	0.442	56	28	65	32.5	0.011
Middle	15	40.5	22	59.5		6	15.8	32	84.2		21	10.5	54	27	
High	0	0	0	0		0	0	4	100		0	0	4	2	

Note: Low QoL = total WHOQOL-BREF score < 50 (0–100 transformed scale). High QoL = total WHOQOL-BREF score ≥ 50. The cut-off of 50 represents the midpoint of the scale and was used solely for the descriptive comparison presented in this table. All subsequent analyses.

**Table 4:** The differences between the participants’ sociodemographic and relevant characteristics based on their quality of life.

Variable		Total											
		Physical			Psychological			Social			Environmental		
		µ	SD	*p*	µ	SD	*p*	µ	SD	*p*	µ	SD	*p*
Age (Y)	18–29	62.17	19.967	0.112	56.37	19.448	0.281	52.77	23.386	0.059	49.01	22.507	0.489
	30–39	60.22	18.909		53.16	18.635		59.59	23.225		53.12	19.339	
	40–49	52.13	23.258		47.43	26.782		45.30	27.047		48.35	25.380	
	50–59	61.08	19.491		53.17	20.511		57.75	26.677		57.58	22.637	
	≥60	67.50	5.745		56.50	15.242		62.50	14.434		59.50	8.266	
Gender	Male	61.82	19.875	0.151	56.80	19.975	0.032	52.51	23.161	0.498	50.95	21.392	0.926
	Female	57.67	20.744		50.44	21.472		54.87	25.744		50.66	22.787	
Marital State	Married	59.09	21.727	0.169	51.86	22.032	0.200	56.53	25.272	0.155	52.80	23.157	0.519
	Single	62.37	18.901		57.03	17.565		52.65	23.410		48.59	20.374	
	Widow	48.50	10.928		46.13	23.216		41.38	23.784		53.25	19.833	
	Divorced	52.70	20.548		47.60	29.964		43.60	22.677		45.60	25.992	
Living Arrangement	Big cities	63.92	20.166	0.000	57.59	20.435	0.000	57.12	23.766	0.007	58.02	19.964	0.000
	towns	56.55	19.088		55.15	22.312		55.60	23.175		56.75	20.191	
	village	45.89	16.872		37.21	17.303		40.21	21.395		39.16	18.476	
	Low social	13.00			13.00			0.00			25.00		
	Camps	56.05	18.701		48.95	18.508		50.48	25.689		32.33	17.432	
Educational Level	Illiterate	31.67	10.970	0.094	37.67	6.506	0.435	29.00	3.464	0.334	23.00	20.224	0.233
	Elementary	61.10	22.270		50.24	21.159		52.19	22.523		51.71	22.918	
	Secondary	61.74	19.506		55.90	20.491		54.83	24.748		51.30	21.787	
	Diploma	55.04	19.841		54.48	19.904		49.70	21.726		47.22	24.125	
	Bachelors	55.72	20.504		49.94	24.158		54.56	29.818		50.97	20.958	
	Masters	72.00	10.392		64.00	17.068		72.00	3.464		68.50	14.434	
	Doctoral	62.67	18.502		54.33	7.506		64.67	13.051		52.00	3.464	
Employment Status	None	59.21	21.624	0.259	51.70	21.597	0.056	53.42	25.249	0.629	50.09	22.044	0.037
	Part-time	56.31	17.197		52.91	17.001		51.43	23.621		45.66	19.909	
	Full time	66.95	16.888		64.55	20.722		57.73	22.442		61.82	23.730	
	Retired	65.00	3.464		60.67	15.631		66.67	14.434		62.67	6.506	
Discrimination	Low	60.29	21.802	0.189	56.01	22.005	0.035	53.88	25.666	0.272	52.71	23.571	0.175
	Medium	56.50	16.539		47.46	16.449		51.52	21.744		46.94	16.068	
	High	69.63	15.766		50.00	23.483		66.50	19.828		43.75	28.080	
Years of Immigration	10 years	52.82	17.190	0.000	50.08	19.258	0.151	46.67	22.584	0.008	40.08	18.634	0.000
	9–5 years	60.03	19.473		53.90	19.732		57.50	23.971		51.50	22.451	
	<5 years	67.89	22.367		57.25	23.995		58.19	25.873		63.65	18.619	
Monthly Family Income	Low	57.13	21.793	0.040	51.50	21.084	0.018	51.41	25.466	0.068	46.17	21.453	0.000
	Middle	62.77	17.355		55.21	19.945		56.29	22.614		56.61	20.763	
	High	76.75	16.256		80.00	19.782		76.50	15.801		81.50	18.230	

**Table 5:** The differences in participants’ sociodemographic and relevant characteristics based on their quality of life, categorized by their country of residence.

Variable		Jordan												Spain											
		Physical			Psychological			Social			Environmental			Physical			Psychological			Social			Environmental		
		µ	SD	*p*	µ	SD	*p*	µ	SD	*p*	µ	SD	*p*	µ	SD	*p*	µ	SD	*p*	µ	SD	*p*	µ	SD	*p*
Age (Y)	18–29	54.26	16.011	0.016	51.66	16.289	0.047	48.90	23.347	0.110	37.12	15.706	0.090	73.17	19.888	0.191	62.92	21.710	0.636	58.14	22.672	0.176	65.53	20.058	0.805
	30–39	55.79	17.366		49.18	15.137		50.89	20.154		42.50	16.745		64.37	19.627		56.87	20.969		67.70	23.259		63.03	16.232	
	40–49	40.13	15.353		37.13	19.252		36.75	23.382		32.13	14.366		60.13	24.424		54.29	29.181		51.00	28.269		59.17	25.542	
	50–59	56.25	10.210		53.25	16.317		51.50	15.801		40.75	19.568		63.50	23.071		53.13	23.388		60.88	31.270		66.00	19.957	
	≥60	66.00	4.243		53.50	13.435		75.00	0.000		59.50	4.950		69.00	8.485		59.50	21.920		50.00	0.000		59.50	13.435	
Gender	Male	52.81	14.66	0.964	50.96	16.60	0.134	50.68	22.94	0.196	40.65	15.68	0.114	75.34	19.11	0.001	65.55	21.57	0.013	55.24	23.53	0.203	66.39	19.55	0.213
	Female	52.65	19.43		45.79	17.15		44.77	21.88		35.47	16.62		61.15	21.06		53.66	23.62		61.87	26.05		61.19	20.49	
Marital State	Married	49.00	18.476	0.195	43.14	19.155	0.013	46.86	22.749	0.630	35.75	19.808	0.201	64.51	21.521	0.020	56.55	22.176	0.440	61.73	25.182	0.314	61.96	19.418	0.639
	Single	55.98	15.749		53.67	14.689		50.27	23.527		40.55	14.050		77.00	17.560		64.71	21.243		58.08	22.687		67.04	20.838	
	Widow	46.20	10.402		40.00	10.464		38.80	16.053		43.80	8.843		52.33	12.897		56.33	37.647		45.67	37.687		69.00	25.000	
	Divorced	50.00	16.269		42.25	12.816		42.00	12.936		26.50	5.745		54.50	24.321		51.17	38.463		44.67	28.668		58.33	26.643	
Living Arrangement	Big cities	51.29	15.140	0.225	53.29	15.639	0.015	46.94	21.073	0.247	41.69	14.728	0.010	69.12	19.735	0.004	59.36	21.947	0.084	61.31	23.655	0.046	64.74	17.887	0.005
	towns	54.70	15.370		50.10	11.090		56.30	18.239		48.30	12.157		58.40	22.921		60.20	29.518		54.90	28.286		65.20	23.541	
	village	46.00	14.885		36.67	13.968		39.27	17.898		40.47	13.543		45.50	25.955		39.25	29.647		43.75	35.075		34.25	33.935	
	Low social	-	-		-	-		-	-		-	-		13.00	.		13.00	.		0.00	.		25.00	.	
	Camps	56.05	18.701		48.95	18.508		50.48	25.689		32.33	17.432		-	-		-	-		-	-		-	-	
Educational Level	Illiterate	28.50	13.435	0.254	37.50	9.192	0.760	28.00	4.243	0.220	15.50	21.920	0.012	38.00	.	0.564	38.00	.	0.410	31.00	.	0.770	38.00	.	0.809
	Elementary	51.29	17.287		44.24	16.943		47.82	19.771		35.76	17.598		67.76	23.102		54.32	23.034		55.16	24.152		62.56	19.700	
	Secondary	54.31	16.840		49.60	17.449		46.40	23.558		36.89	14.898		68.71	19.413		61.81	21.522		62.73	23.399		64.81	18.331	
	Diploma	50.62	13.781		48.08	13.871		42.23	16.833		35.85	11.503		60.80	25.376		62.80	23.994		59.40	24.309		62.00	28.577	
	Bachelor’s	51.61	18.118		50.83	19.669		53.56	26.351		43.56	16.621		61.00	22.800		48.79	29.720		55.86	34.769		60.50	22.633	
	Master’s	69.00	10.392		58.33	15.631		73.00	3.464		64.33	14.434		81.00	.		81.00	.		69.00	.		81.00	.	
	Doctoral	53.50	13.435		50.00	0.000		62.50	17.678		50.00	0.000		81.00	.		63.00	.		69.00	.		56.00	.	
Employment Status	None	49.86	17.280	0.086	46.08	17.802	0.242	46.84	23.352	0.348	35.32	15.245	0.021	66.86	21.906	0.647	56.30	23.387	0.079	58.81	25.609	0.950	62.18	19.239	0.432
	Part-time	55.50	15.613		53.42	14.191		47.85	21.273		41.00	14.439		58.67	22.062		51.44	24.409		61.78	28.195		59.11	27.547	
	Full time	62.00	13.332		52.78	17.218		52.11	21.257		48.00	22.028		70.38	18.693		72.69	19.444		61.62	23.240		71.38	20.484	
	Retired	66.00	4.243		53.50	13.435		75.00	0.000		59.50	4.950		63.00	.		75.00	.		50.00	.		69.00	.	
Discrimination	Low	52.06	17.468	0.308	50.14	16.825	0.422	47.46	22.587	0.230	38.17	16.857	0.396	68.52	22.693	0.137	61.87	24.955	0.038	60.29	27.083	0.602	67.25	20.159	0.005
	Medium	52.38	14.397		46.23	15.085		46.69	22.590		40.69	11.902		60.32	17.708		48.61	17.821		56.00	20.305		52.75	17.407	
	High	64.00	17.986		42.40	27.410		65.00	19.183		30.00	25.817		79.00	3.464		62.67	6.506		69.00	25.000		66.67	12.897	
Years of Immigration	10 years	51.89	15.840	0.701	48.82	18.294	0.927	45.88	21.951	0.387	37.48	16.438	0.494	60.38	25.923	0.507	60.38	24.876	0.961	53.13	28.063	0.630	61.25	22.927	0.664
	9–5 years	53.81	19.201		49.00	14.674		52.03	23.661		39.29	15.998		64.97	18.461		57.79	22.401		61.85	23.615		61.21	22.246	
	<5 years	58.25	13.793		45.50	13.178		54.75	24.636		47.00	14.989		68.62	22.805		58.13	24.467		58.45	26.171		64.91	18.369	
Monthly Family Income	Low	50.57	16.627	0.092	47.57	16.329	0.371	44.92	21.344	0.062	34.83	15.275	0.003	64.26	24.494	0.362	55.76	24.705	0.122	58.47	27.794	0.384	58.50	20.450	0.010
	Middle	56.43	16.628		50.73	18.012		53.62	23.817		44.54	16.120		68.95	15.939		59.58	20.983		58.89	21.373		68.37	17.936	
	High	-	-		-	-		-	-		-	-		76.75	16.256		80.00	19.782		76.50	15.801		81.50	18.230	

Note: For statistically significant ANOVA results (*p* < 0.05), post hoc LSD comparisons were conducted. Significant pairwise differences (*p* < 0.05) are indicated in the text (Section 3.4).

## Data Availability

The data that support the findings of this study are available from the corresponding authors upon reasonable request due to privacy.

## Abbreviations

The following abbreviations are used in this manuscript:

ANOVA	Analysis of Variance
BREF	Brief Version
CI	Confidence Interval
ID	Identification Number
LSD	Least Significant Difference
QoL	Quality of Life
SD	Standard Deviation
SPSS	Statistical Package for the Social Sciences
UNHCR	United Nations High Commissioner for Refugees
WHO	World Health Organization
WHOQOL-BREF	World Health Organization Quality of Life—Brief Questionnaire
